# Single-Incision Laparoscopic Totally Extraperitoneal (SILS-TEP) Repair of a Fat Plug-Induced Obturator Hernia With Neuralgia in a Middle-Aged Male Patient

**DOI:** 10.7759/cureus.94981

**Published:** 2025-10-20

**Authors:** Atsushi Gakuhara, Ryugo Teranishi, Kurumi Tsuchihashi, Soichiro Minami, Yutaka Kimura

**Affiliations:** 1 Department of Gastroenterological Surgery, Kindai University Nara Hospital, Ikoma, JPN

**Keywords:** fat plug, male, neuralgia, obturator hernia, sils (single incision laparoscopic surgery), tep

## Abstract

Obturator hernia is a rare type of pelvic hernia most commonly seen in elderly women. We report a case of a middle-aged man who presented with obturator neuralgia caused by an obturator hernia containing only preperitoneal fat without bowel involvement. The patient underwent single-incision laparoscopic totally extraperitoneal (SILS-TEP) repair, which promptly relieved his symptoms with no recurrence. This case highlights that a fat plug-type obturator hernia can cause neuralgia and demonstrates the efficacy of SILS-TEP as a minimally invasive treatment option.

## Introduction

An obturator hernia is often diagnosed late due to its nonspecific presentation, such as vague groin pain, medial thigh discomfort, or lower abdominal pain, and is therefore considered difficult to diagnose. Typically, bowel herniates through the obturator canal, producing medial thigh pain known as the Howship-Romberg sign [1,2]. Classically, an obturator hernia presents with small-bowel obstruction requiring urgent intervention [1]. Rarely, however, only preperitoneal fat protrudes through the canal, compressing the obturator nerve and producing neuralgic symptoms [3,4]. Such a fat plug-type obturator hernia, possibly representing a precursor stage of true hernia formation, has been scarcely reported [2]. We report a rare case of a fat plug-type obturator hernia in a middle-aged male patient successfully treated with single-incision laparoscopic totally extraperitoneal (SILS-TEP) repair.

## Case presentation

A 53-year-old man presented with a two-month history of dull pain radiating from the left lower abdomen to the medial thigh. The pain worsened with trunk rotation and was occasionally sharp and electric in nature. His past medical history included coronary artery bypass grafting, type 2 diabetes mellitus, and left inguinal hernia repair in his 20s.

**Figure 1:**
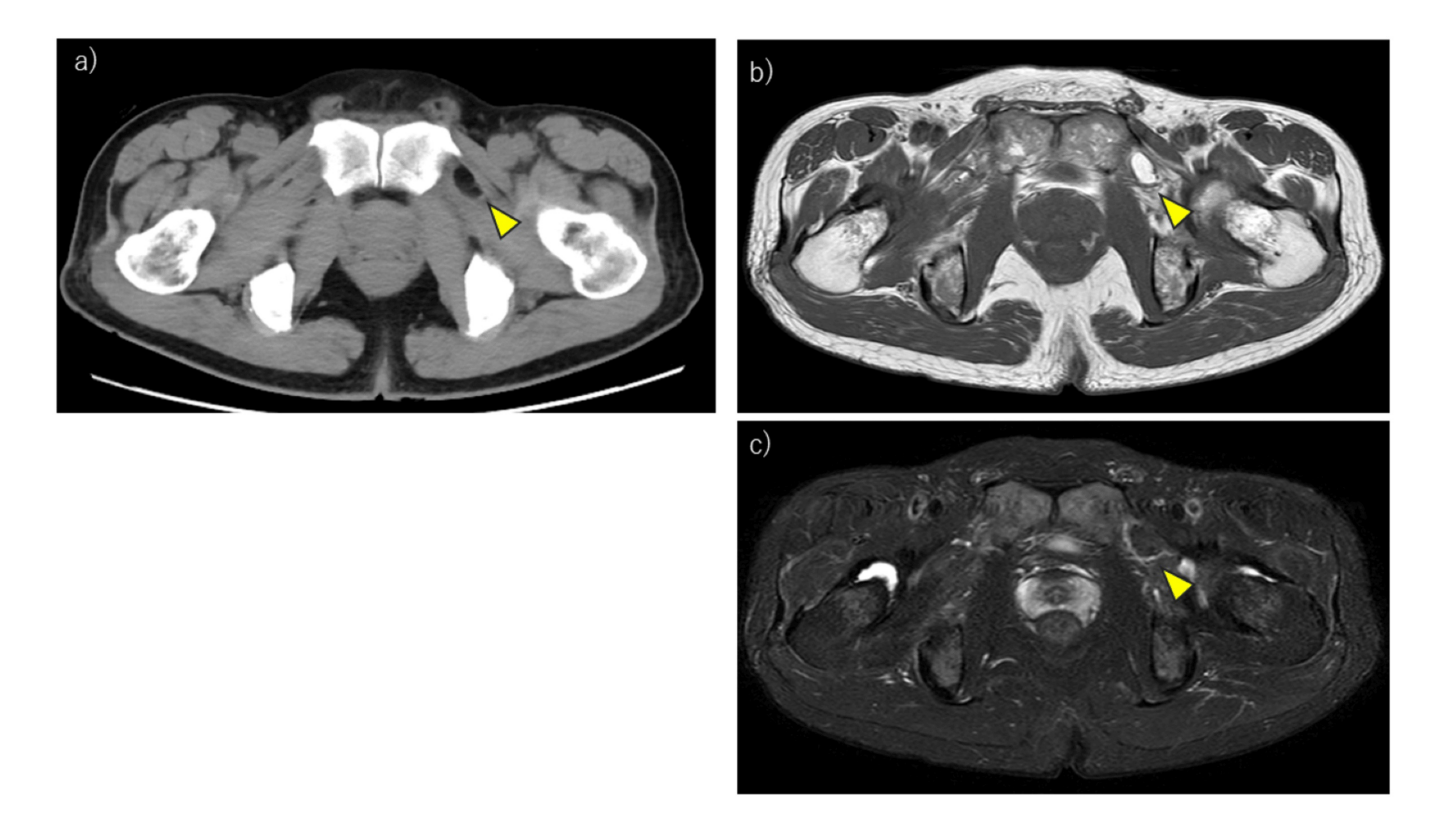
Preoperative imaging (a) CT showing fat herniation into the left obturator canal (yellow arrowhead). (b) MRI T2-weighted image showing high signal intensity in the corresponding area (yellow arrowhead). (c) Fat-suppressed MRI demonstrating signal suppression of the same lesion (yellow arrowhead).

Abdominal CT revealed herniation of fat into the left obturator canal (Figure 1a). MRI showed high signal intensity on T2-weighted images (Figure 1b) and suppression on fat-suppressed images (Figure 1c), confirming the lesion as fat tissue without bowel involvement. These findings suggested compression of the obturator nerve by herniated fat. Based on the clinical symptoms and imaging, an obturator hernia was diagnosed, and surgical repair was indicated despite the atypical age and sex of the patient.

A SILS-TEP repair was performed to avoid entering the peritoneal cavity, as compared to the transabdominal preperitoneal (TAPP) approach. A 25 mm infraumbilical incision was made, and the anterior rectus sheath was incised for approximately 30 mm. The space between the rectus muscle and the posterior sheath was bluntly dissected. A Lap-Protector Mini (Hakko Co., Nagano, Japan) was placed to maintain this working space, and three 5 mm trocars were inserted through a single-port access device (EZ Access; Hakko Co., Nagano, Japan). Carbon dioxide insufflation was maintained at 10 mmHg to facilitate blunt dissection of the preperitoneal space under laparoscopic visualization.

**Figure 2:**
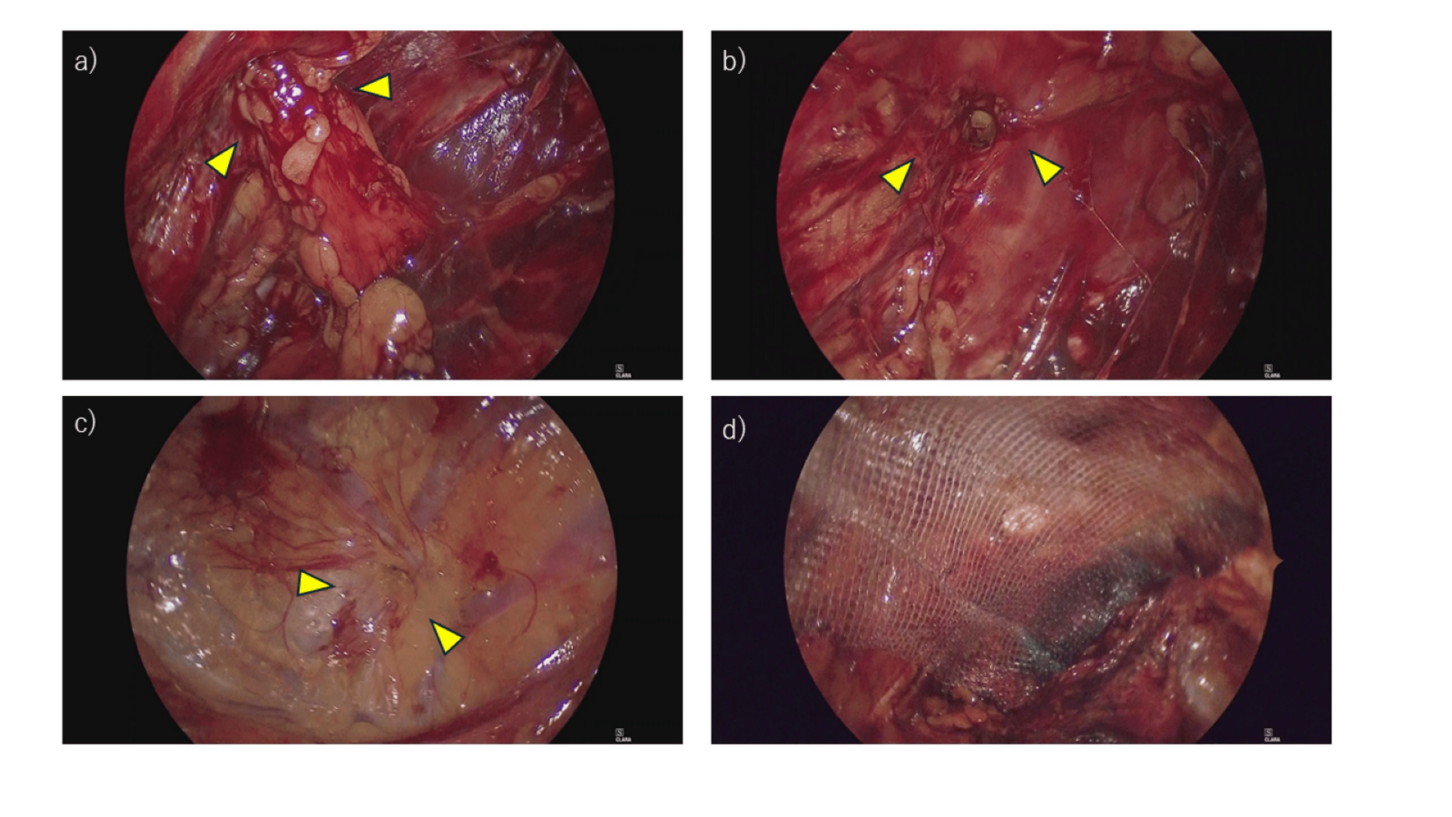
Intraoperative findings (a) Fat herniating into the left obturator canal observed in the preperitoneal space (yellow arrowheads). (b) Hernia defect after removal of the fat plug (yellow arrowheads). (c) Right obturator canal showing no abnormality (yellow arrowheads). (d) Placement of a self-fixating mesh covering the left obturator canal.

On the left side, dissection was extended laterally to the anterior superior iliac spine and caudally until the obturator foramen was identified. On the right side, the dissection was continued until the right obturator canal was clearly visualized. Fat protruding into the left obturator canal was identified (Figure 2a), carefully dissected, and removed (Figure 2b). The obturator nerve was identified adjacent to the herniated fat within the obturator canal. It was preserved without injury, and care was taken to avoid traction or thermal damage during dissection. The right obturator canal was unremarkable (Figure 2c).

A self-fixating polypropylene mesh (15 × 10 cm) was positioned to cover the entire myopectineal orifice, including Cooper’s ligament; the direct, indirect, and femoral spaces; and the obturator canal, with approximately a 3 cm overlap beyond the defect margins. The mesh was smoothly adapted to the pelvic contour without fixation (Figure 2d).

The operative time was 105 minutes, blood loss was 3 mL, and there were no intraoperative complications. The patient’s pain resolved immediately after surgery, and he was discharged on postoperative day 2. A quantitative pain score such as the visual analog scale (VAS) was not recorded; however, the neuralgia disappeared completely and did not recur during the three-month follow-up period. As the patient lived far from our institution, postoperative follow-up was completed at three months.

## Discussion

An obturator hernia is extremely rare, accounting for 0.05%-1.4% of all abdominal wall hernias, with most cases occurring in thin, elderly women [5]. This gender and age distribution is attributed to the larger obturator canal in women and the age-related atrophy of pelvic tissues [1,6]. Conversely, an obturator hernia in men is uncommon, and cases in middle-aged men are particularly rare [1,7]. Reports of fat plug-type hernias causing neuralgia without bowel involvement are limited [3,4].

The obturator canal transmits the obturator nerve and vessels. Compression of the obturator nerve, originating from L2-L4, produces characteristic neuralgia radiating to the medial thigh, recognized as the Howship-Romberg sign [1,2]. In our case, despite the absence of bowel herniation, localized neuralgia corresponded to nerve compression by fat, representing a clinically and anatomically significant presentation.

Diagnosis of an obturator hernia relies on imaging since physical examination is often unrevealing. CT demonstrates herniation of fat or bowel through the obturator canal, while MRI provides further information about nerve compression and surrounding soft tissues [2,6]. In this case, the consistency between imaging findings and clinical symptoms strongly suggested fat plug-induced obturator neuralgia.

Fat plug herniation, also referred to as a “pre-hernial condition,” differs from true bowel-containing hernias [2]. Even without bowel involvement, nerve compression by herniated fat can cause severe neuralgia. Perry and Hantes described seven women with obturator neuralgia due to type I obturator hernia who improved after laparoscopic reduction of the fat tag with mesh overlay [8]. Petrushnko et al. also reported successful laparoscopic TEP repair for an obturator hernia [3].

Surgical repair remains the standard treatment for an obturator hernia. While open laparotomy was historically used, laparoscopic approaches such as TAPP and TEP are now commonly advocated and associated with faster recovery; mesh reinforcement appears to reduce recurrence [6]. In this case, SILS-TEP was selected for its minimal invasiveness and cosmetic advantage. The patient’s pain resolved immediately, and he was discharged after a short hospital stay.

Thus, SILS-TEP appears to be a safe and effective minimally invasive treatment option for obturator hernia, even in rare fat plug-type cases presenting with neuralgia. Bilateral cases have also been successfully diagnosed and treated laparoscopically [4]. This has been supported by a dedicated review of the laparoscopic approach [9]. Technical details and step-by-step considerations for TEP repair in challenging patients have also been described [6]. A contemporary review summarizes diagnostic strategies and management considerations for an obturator hernia [7].

Only a few cases of obturator hernia repaired by the SILS-TEP approach have been reported. Wakasugi et al. first described this technique in 2015 as the initial case of SILS-TEP for obturator hernia, demonstrating its technical feasibility and good visualization of the obturator canal [10]. In a later series of over 300 SILS-TEP procedures, only two cases involved an obturator hernia, underscoring the rarity of this approach [11].

The main challenges of SILS-TEP are limited triangulation [12] and the risk of peritoneal tearing in the narrow preperitoneal space. In our experience, maintaining adequate insufflation pressure, keeping dissection close to the posterior rectus sheath, and early identification of Cooper’s ligament and the obturator canal were key to ensuring safe and accurate mesh placement. These technical considerations may assist surgeons in safely performing SILS-TEP in similar rare cases.

## Conclusions

A fat plug-type obturator hernia can cause neuralgia without bowel involvement and is diagnosed by linking medial thigh pain with CT/MRI findings. Though rare in middle-aged men, SILS-TEP provided immediate symptom relief and early recovery, supporting it as a safe and minimally invasive treatment.
